# Racial Disparities in the Utilization and Outcomes of Temporary Mechanical Circulatory Support for Acute Myocardial Infarction-Cardiogenic Shock

**DOI:** 10.3390/jcm10071459

**Published:** 2021-04-02

**Authors:** Rahul Vojjini, Sri Harsha Patlolla, Wisit Cheungpasitporn, Arnav Kumar, Pranathi R. Sundaragiri, Rajkumar P. Doshi, Allan S. Jaffe, Gregory W. Barsness, David R. Holmes, S. Tanveer Rab, Saraschandra Vallabhajosyula

**Affiliations:** 1 Department of Cardiovascular Medicine, Mayo Clinic, Rochester, MN 55905, USA; drrahul148@gmail.com (R.V.); Jaffe.Allan@mayo.edu (A.S.J.); Barsness.Gregory@mayo.edu (G.W.B.); Holmes.David@mayo.edu (D.R.H.); 2 Department of Cardiovascular Surgery, Mayo Clinic, Rochester, MN 55905, USA; Patlolla.SriHarsha@mayo.edu; 3 Division of Nephrology and Hypertension, Department of Medicine, Mayo Clinic, Rochester, MN 55905, USA; wcheungpasitporn@gmail.com; 4 Division of Cardiovascular Medicine, Department of Medicine, Emory University School of Medicine, Atlanta, GA 30322, USA; arnav.kumar@emory.edu; 5 Jen Care Senior Medical Center, ChenMed, Morrow, GA 30260, USA; drpranathi99@gmail.com; 6 Department of Medicine, University of Nevada Reno School of Medicine, Reno, NV 89557, USA; raj20490@gmail.com; 7 Section of Interventional Cardiology, Division of Cardiovascular Medicine, Department of Medicine, Emory University School of Medicine, Atlanta, GA 30322, USA; srab@emory.edu

**Keywords:** cardiogenic shock, race, healthcare disparities, mechanical circulatory support, acute myocardial infarction, minorities

## Abstract

Racial disparities in utilization and outcomes of mechanical circulatory support (MCS) in patients with acute myocardial infarction-cardiogenic shock (AMI-CS) are infrequently studied. This study sought to evaluate racial disparities in the outcomes of MCS in AMI-CS. The National Inpatient Sample (2012–2017) was used to identify adult AMI-CS admissions receiving MCS support. MCS devices were classified as intra-aortic balloon pump (IABP), percutaneous left ventricular assist device (pLVAD) or extracorporeal membrane oxygenation (ECMO). Self-reported race was classified as white, black and others. Outcomes included in-hospital mortality, hospital length of stay and discharge disposition. During this period, 90,071 admissions were included with white, black and other races constituting 73.6%, 8.3% and 18.1%, respectively. Compared to white and other races, black race admissions were on average younger, female, with greater comorbidities, and non-cardiac organ failure (all *p* < 0.001). Compared to the white race (31.3%), in-hospital mortality was comparable in black (31.4%; adjusted odds ratio (aOR) 0.98 (95% confidence interval (CI) 0.93–1.05); *p* = 0.60) and other (30.2%; aOR 0.96 (95% CI 0.92–1.01); *p* = 0.10). Higher in-hospital mortality was noted in non-white races with concomitant cardiac arrest, and those receiving ECMO support. Black admissions had longer lengths of hospital stay (12.1 ± 14.2, 10.3 ± 11.2, 10.9 ± 1.2 days) and transferred less often (12.6%, 14.2%, 13.9%) compared to white and other races (both *p* < 0.001). In conclusion, this study of AMI-CS admissions receiving MCS devices did not identify racial disparities in in-hospital mortality. Black admissions had longer hospital stay and were transferred less often. Further evaluation with granular data including angiographic and hemodynamic parameters is essential to rule out racial differences.

## 1. Introduction

Around 5–10% of all acute myocardial infarction (AMI) admissions are complicated by cardiogenic shock (CS). These admissions have nearly 30–50% in-hospital mortality [1,2]. In addition to restoring coronary perfusion with early revascularization, temporary mechanical circulatory support (MCS) devices are frequently used for hemodynamic support in this population [3]. Decisions to employ temporary MCS device selection are based on several factors including the severity of CS, level of hemodynamic support required, patient comorbidities, and technical limitations [4,5]. Due to the need for significant resource allocation and technical support, it is conceivable that health care inequalities due to patient demographics such as age, sex and race may arise in this population, similar to that in other conditions in acute cardiovascular care [6,7,8].

Studies have in fact demonstrated racial, regional, sex based and age related disparities in treatment and outcomes of AMI-CS patients [2,9,10,11] Reports focusing on racial differences in AMI-CS population have shown that non-white patients are associated with lower use of guideline directed therapies and worse outcomes [9,12] However, these reports are either more than a decade old or are non-specific and include patients with CS secondary to multiple causes. [9,12] Therefore, we evaluated racial disparities in the utilization and outcomes of temporary MCS specific for AMI-CS using large-scale contemporary data. We hypothesized that racial minorities would have less frequent MCS use and would have worse outcomes as compared to white admissions. Using a six-year national database, we assessed differences between races in the utilization and outcomes of MCS devices in AMI-CS. We also evaluated demographics, clinical characteristics and management strategies of AMI-CS stratified by race.

## 2. Methods

The National (Nationwide) Inpatient Sample (NIS) is an all-payer administrative database of inpatient hospital stays in the United States, created as part of the Healthcare Quality and Utilization Project (HCUP), sponsored by the Agency for Healthcare Research and Quality. It includes a 20% stratified sample of community hospitals [13]. Each record includes information on patient and hospital characteristics, data on principal diagnosis, in-hospital procedures, and up to 39 secondary diagnoses. Information is captured for a given admission and not for individual patients. The data is publicly available via the HCUP-NIS database and hence Institutional Review Board approval was not obtained.

Using the HCUP-NIS data from 1 January 2012 to 31 December 2017, we identified adult (>18 years) admissions with AMI in the primary diagnosis field using International Classification of Diseases 9.0 Clinical Modification (ICD-9CM) codes 410.x and ICD-10CM I21.x − 22.x. Admissions with a secondary diagnosis of CS were identified with ICD-9CM 785.51, or ICD-10CM R57.0 [11,14,15]. The administrative codes for CS have high positive predictive value (>90%) and specificity (>95%) but low sensitivity (>50%) [16,17]. We identified use of MCS devices: extracorporeal membrane oxygenation (ECMO), intra-aortic balloon pump (IABP) and percutaneous left ventricular assist device (Impella or TandemHeart) (pLVAD) using respective ICD-CM and ICD-PCS codes (Supplementary Table S1). Our analysis focused on admissions receiving these devices in our study [18,19,20]. For the purposes of this analysis, race was classified as white, black and others (Hispanic, Asian or Pacific Islander, Native American, Others). Coding for race in NIS combines ‘race’ and ‘ethnicity’ provided by the data source into one data element (RACE). If both ‘race’ and ‘ethnicity’ were available, ethnicity was preferred over race in setting the HCUP value for ‘RACE’ [13]. In the NIS database, racial classification was missing for approximately 23% of the sample in 2000 and improved over the years with 3.6% missing in the HCUP-NIS 2017 data. During the initial years of NIS, certain states have withheld racial/ethnic classification. Hence it is unlikely that data was missing completely at random [21]. Admissions with missing race/ethnicity were excluded from the analysis. The HCUP-NIS provides a quartile classification of the estimated median household income of residents in the patient’s ZIP Code. The quartiles indicate the poorest to wealthiest populations. These values are derived from ZIP Code-demographic data obtained from Claritas [13]. We used Deyo’s modification of the Charlson Comorbidity Index to estimate comorbidity burden [22]. Demographic characteristics including age, sex, race, hospital characteristics, acute organ failure, MCS, cardiac procedures, and noncardiac procedures were identified using methodologies similar to prior work from our group (Supplementary Table S1).

The primary outcome of interest was racial disparities in the in-hospital mortality of AMI-CS admissions receiving MCS devices. The secondary outcomes included racial disparities in mechanical circulatory support (MCS) use, palliative care services, hospitalization costs, hospital length of stay, and discharge disposition. Multiple sub-group analyses were performed to confirm the results of the primary analysis stratifying the population by sex (male/female), type of AMI (ST-segment elevation (STEMI) vs. non-ST-segment elevation (NSTEMI)), receipt of PCI, presence of cardiac arrest and timing of MCS use (early/delayed). 

### Statistical Analysis

As per HCUP-NIS recommendations, we used survey procedures with discharge weights provided from the HCUP-NIS database to generate national estimates [23]. Categorical variables were compared with Chi-square test whereas *t*-tests were used for continuous data. Trends over time were analyzed using multivariable logistic regression (referent year 2012). Trends and outcomes were analyzed in a univariable analysis and represented as odds ratio (OR) with 95% confidence interval (CI). Multivariable logistic regression analysis was performed including age, sex, primary payer status, socio-economic stratum, hospital characteristics, comorbidities, acute organ failure, AMI-type, cardiac arrest, cardiac procedures, and non-cardiac procedures as variables for assessing temporal trends analyses and in-hospital mortality. Purposeful selection of clinically and statistically (liberal threshold of *p* < 0.20 in univariate analysis) relevant variables was conducted for multivariable regression analysis. A two-tailed *p* < 0.05 was considered statistically significant. 

Pertinent considerations related to research design, data interpretation, and data analysis of the HCUP-NIS database were reviewed and addressed [23]. These included not assessing individual hospital-level volumes, treating each entry as an ‘admission’ as opposed to an individual patient, evaluating inpatient factors, since the database does not include outpatient data, and using previously validated administrative codes or those used for similar diagnosis in prior studies. All data was analyzed using SPSS v25.0 (IBM Corp., Armonk, NY, USA).

## 3. Results 

**Figure 1:**
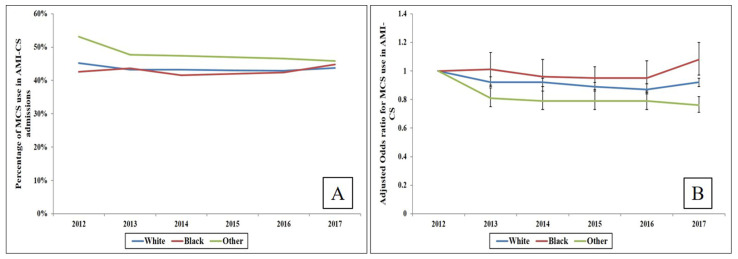
Trends in MCS use among race sub-groups of AMI-CS admissions. (**A**) Unadjusted trends in MCS use among racial sub-groups of AMI-CS (*p* < 0.05 for temporal trend in white and other races); (**B**) Adjusted multivariable logistic regression for MCS use among race sub-groups of AMI-CS (referent year 2012); adjusted for age, sex, household income status, primary payer, comorbidity, and hospital characteristics (*p* < 0.001 for temporal trend). Abbreviations: AMI: acute myocardial infarction; CS: cardiogenic shock; MCS: mechanical circulatory support

A total of 203,905 AMI-CS admissions were identified between 1 January 2012 and 31 December 2017, of which 90,071 (44.2%) were supported with MCS. White, black and other races constituted 66,314 (73.6%), 7440 (8.3%) and 16,317 (18.1%) admissions, respectively. Black admissions received less frequent MCS support compared to white and other races (42.6% vs. 43.5% vs. 48.0%, *p* < 0.001). Unadjusted analysis of MCS device use over the years in AMI-CS admissions indicated a higher use but declining trend in other races while white and black races had a relatively steady trend over the six-year period (Figure 1A). Adjusted analysis revealed a relatively stable trend with a mild increase in the black race and decrease in other races (Figure 1B). Compared to white and other races, black race admissions were on average younger, female and from the lowest income quartile, with greater comorbidities, higher rates of NSTEMI presentation, and non-cardiac organ failure (Table 1). Other race admissions received higher rates of coronary angiography, invasive and non-invasive mechanical ventilation while whites received higher rates of PCI (Table 1).

**Figure 2:**
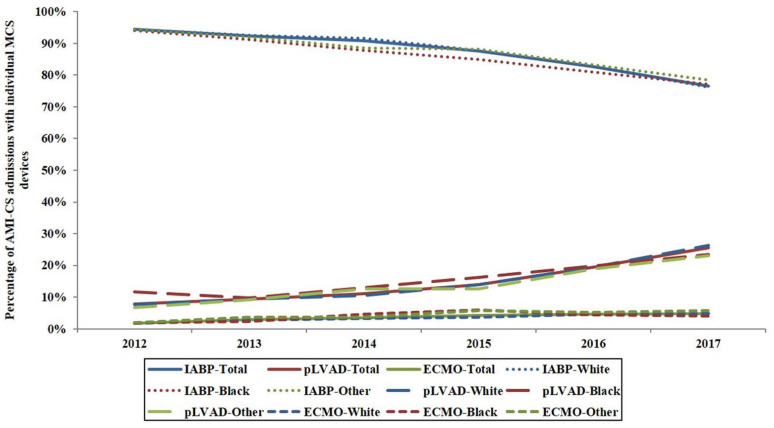
Trends in the use of MCS devices in race sub-groups of AMI-CS admissions. All *p* < 0.001 for temporal trend. Abbreviations: AMI: acute myocardial infarction; CS: cardiogenic shock; ECMO: extracorporeal membrane oxygenation; IABP: intra-aortic balloon pump; MCS: mechanical circulatory support; pLVAD: percutaneous left ventricular assist device.

In the cohort requiring MCS support, IABP was used in 73,979 (82.1%), pLVAD in 9890 (11.0%), ECMO in 1386 (1.5%), and ≥2 MCS devices were used in 4816 (5.4%). The trends in use of these devices from 2012 to 2017 in sub-groups of race were similar in white, black and other races (Figure 2). Blacks receiving any of these devices were on average younger, more likely to be female, and from a lower socio-economic stratum compared to white and other races (Supplementary Table S2). Other race admissions receiving IABP, pLVAD and ≥2MCS devices on average had higher rates of acute organ failure and greater use of invasive mechanical ventilation compared to black and white races. A lower rate of PCI use was noticed in other race admissions receiving IABP while a higher rate of PCI use was noted in other race admissions receiving ECMO compared to whites and blacks (Supplementary Table S2).

**Figure 3:**
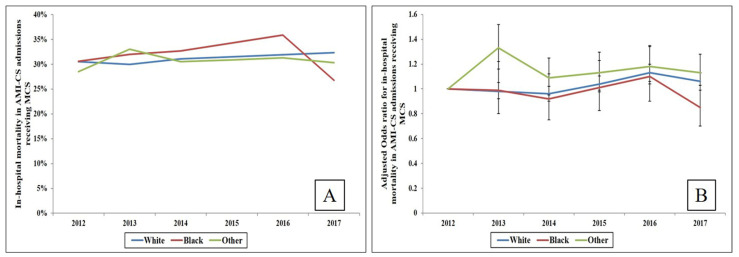
Trends of in-hospital mortality in race sub-groups of AMI-CS admissions receiving MCS. (**A**) Unadjusted in-hospital mortality in race sub-groups of AMI-CS admissions supported with MCS (*p* < 0.001 for temporal trend only for white); (**B**) Adjusted temporal trends of in-hospital mortality in race sub-groups of AMI-CS admissions receiving MCS (referent year 2012); adjusted for age, sex, household income status, primary payer, comorbidity, hospital characteristics, AMI type, acute organ failure, cardiac arrest, cardiac and non-cardiac procedures (*p* < 0.05 for trend over time for white and other races). Abbreviations: AMI: acute myocardial infarction; CS: cardiogenic shock; MCS: mechanical circulatory support.

**Figure 4:**
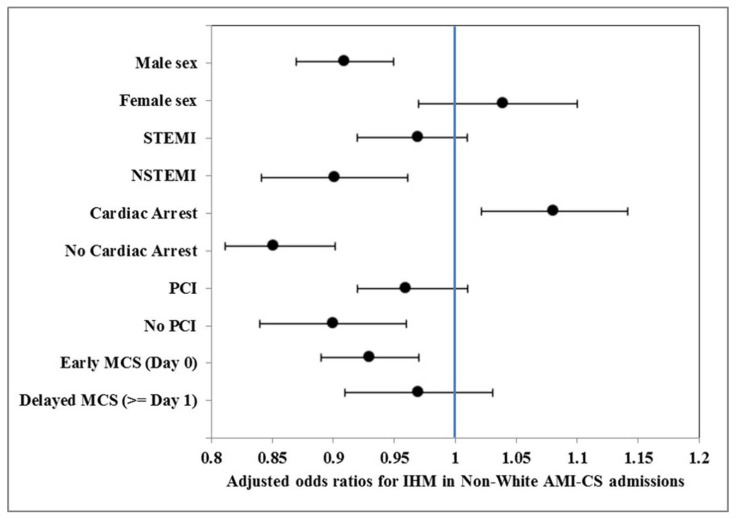
Multivariable logistic regression analysis comparing in-hospital mortality in non-whites to white race in sub-groups of AMI-CS admissions receiving MCS. Legend: The figure shows multivariable adjusted odds ratios (95% confidence intervals) * for in-hospital mortality in non-whites compared to white race in sub-groups of sex, presence of cardiac arrest, type of AMI, timing of MCS placement and receipt of PCI. * Adjusted for patient and hospital characteristics, admission year, comorbidity, presence of cardiac arrest, type of AMI-CS, acute organ failure, cardiac and non-cardiac procedures. Abbreviations: AMI: acute myocardial infarction; CS: cardiogenic shock; IHM: in-hospital mortality; MCS: mechanical circulatory support; NSTEMI: non-ST-elevation myocardial infarction; PCI: percutaneous coronary intervention; STEMI: ST-elevation myocardial infarction.

In-hospital mortality was lower in other races (30.2%) compared to white (31.3%) and black (31.4%), but adjusted analyses revealed comparable in-hospital mortality risk among all three racial categories (Supplementary Table S3). Temporal trends analysis showed a relatively stable trend across all racial sub-groups during this six-year period (Figure 3A,B). In stratified sub-group analyses, compared to white race, a higher risk of mortality was identified in non-white admissions with cardiac arrest, but no other significant disparities were noted (Figure 4). Black admissions had longer length of hospital stay and were more likely to be discharged to a skilled nursing facility compared to white and other races (Table 2).

AMI-CS admissions receiving ECMO support had higher in-hospital mortality, longer length of hospital stay, higher rates of palliative care consultation, higher hospitalization costs and less frequent discharges to home compared to those receiving IABP or pLVAD across all races (Supplementary Table S4). Other races had lower unadjusted in-hospital mortality in those receiving IABP and pLVAD but higher in-hospital mortality in those receiving ECMO (Supplementary Table S4). After adjusting for potential confounders, other race had lower in-hospital mortality risk in those receiving IABP alone (OR 0.92 (95% CI 0.87–0.97); *p* = 0.001) and higher in-hospital mortality risk among those receiving ECMO alone (OR 2.01 (95% CI 1.43–2.81); *p* < 0.001) with comparable risk of mortality among those receiving pLVAD alone (OR 0.97 (95% CI 0.86–1.11); *p* = 0.70) compared to white race. Black race had greater adjusted in-hospital mortality risk in those receiving ECMO alone (OR 2.49 (95% CI 1.50–4.13); *p* < 0.001) and comparable mortality in those receiving IABP (OR 0.94 (95% CI 0.87–1.01]; *p* = 0.08) or pLVAD (OR 1.07 (95% CI 0.91–1.26); *p* = 0.41) compared to white race (data not shown). Among individuals receiving ≥2 MCS devices there were no differences in adjusted risk of in-hospital mortality between races.

## 4. Discussion

In this study evaluating racial differences in utilization and outcomes of MCS in AMI-CS admissions, we noted the following important findings. Black admissions received MCS less frequently compared to people of white and other races; however, there were no differences in the risk of in-hospital mortality across all three racial categories. A consistent decline in the use of an IABP with an increase in pLVAD and ECMO were noted during the study period across all racial categories consistent with larger national trends. Black admissions had significantly longer lengths of hospital stay and were discharged home less frequently. Though minor differences in in-hospital mortality were noted between the different MCS device categories, there was no consistent signal towards racial disparities in AMI-CS receiving MCS during the study period.

In patients with AMI-CS, sex, race, socio-economic, hospital-level and patient demographic factors continue to influence outcomes [2,10,11,24,25]. In comparison to AMI, data on racial disparities in AMI-CS are scarce. Using the SHOCK (SHould we emergently revascularize Occluded Coronaries for cardiogenic shocK) Registry data from 1993–1997, Palmeri et al. showed significant racial and ethnic differences in the management and outcomes of AMI-CS patients [9]. They reported under-utilization of coronary angiography, PCI, IABP, and fibrinolytics, while attributing the lower rates of these procedures to the nearly two-fold increase in in-hospital mortality in Hispanics. Indeed, after adjusting for these differences, race was no longer associated with higher mortality [9]. Further, this study was conducted in the era when revascularization for AMI-CS was not considered universally, and the IABP was the only available MCS device. Subsequently, using STEMI-CS data, Kolte et al. reported a higher incidence of CS in the Asian-Pacific islander racial sub-group with lower utilization of PCI and IABP in the African American population. They also found higher in-hospital mortality among Hispanics compared to the white race in AMI-CS hospitalizations [26]. Most recently, Kim et al. identified underutilization of MCS devices and increased odds of in-hospital mortality in black patients compared to other races in an all-comer CS population [12].

Unlike these studies, our study specifically observed AMI-CS admissions receiving MCS during a contemporary six-year period. Despite differences in baseline characteristics, patient demographics and frequency of MCS support, there were no apparent differences in risk of in-hospital mortality across all three racial categories. This is an important observation because racial minorities were consistently reported to have the higher incidence of AMI-CS and its associated mortality [26]. Potential explanation for the difference in the present study may be the focus on the subset of AMI-CS receiving an MCS, stronger adherence to the treatment protocols, improved patient selection strategies over time and early use of revascularization and mechanical circulatory support [26,27,28,29]. However, it is important to note that in comparison to an all-comer CS population wherein racial disparities might still exist in the contemporary era, this was a selected group of AMI-CS admissions wherein MCS was implanted [26]. It is conceivable that patients that did not receive MCS may be systematically different from those that do, including for reasons relating to racial bias [12]. The lower use of MCS in the black race might allude to a bias in implantation only when they were significantly sicker compared to the other races; however, the fact that their outcomes were similar in comparison to other races is reassuring. When stratified by MCS type, consistent with the previous literature, we noted decline in the use of an IABP with an increase in pLVAD and ECMO during the study period [20,30]. It is possible that the superior hemodynamic assistance and biventricular support of these devices over the IABP [20,31] might help offset the greater severity and higher comorbidity in the racial minorities. But this benefit of the newer devices needs to be balanced against the potential risks from the use of these devices [32]. Since our data is observational in nature, these findings are subject to selection bias with potentially sicker patients with more compromised hemodynamics undergoing implantation of these devices. In the present study, white admissions more often presented with STEMI whereas NSTEMI-CS was more frequent among black admissions. A recent study has shown that, between 1996 and 2014, black patients experienced a shift from out-of-hospital STEMI deaths to hospitalized STEMIs and a shift from STEMIs to NSTEMIs by 2014 [33]. Consistent with this trend, our study period from 2012–2017 also shows more NSTEMI-CS in black admissions. A reduction in barriers related to healthcare access and increases in cardioprotective medications, or improved treatment of CVD risk factors for blacks may also be the cause of lower STEMI episodes in blacks [33].

By stratified sub-group analyses, we noted black and other races had higher in-hospital mortality in those receiving ECMO alone. Apart from a higher comorbidity burden, lower socio-economic strata and lack of a well-defined exit strategy in the non-white population, cultural and religious beliefs, treatment preferences and lesser utilization of palliative care services among racial minorities may also have contributed to this disparity [30,34,35]. This alludes to the unfulfilled need to address socio-economic status prior to careful patient selection for ECMO, especially in the setting of AMI-CS. Lastly in the current study when compared to white race, we identified higher risk of mortality in non-white admissions with cardiac arrest. This is consistent with existing literature on racial differences in outcomes with an all-comer cardiac arrest population [36,37,38]. Quality and availability of care, specifically the receipt of coronary angiography and PCI in the acute setting and the social landscape and availability of long-term care, are major determinants of clinical outcomes in this population [36,39,40,41]. This is of particular concern in racial minorities with AMI-CS, as cardiac arrest and its post-discharge disability can contribute to earlier and higher withdrawal of care.

## 5. Limitations

The HCUP-NIS database has several quality control measures to mitigate potential errors. However, this study has limitations inherent to use of secondary data. Use of previously validated administrative codes helps reduce errors with codes in the study. Unavailability of essential data including left ventricular ejection fraction, severity of coronary artery disease, echocardiography, angiography and hemodynamic parameters limits assessments of disease severity. When MCS devices/procedures were used/performed on the same day, we considered those as concomitant. The dynamic nature of the AMI-CS disease process and the lack of granularity in timing of MCS use prevents the discernment of an exact sequence of events. Evaluations of total or individual duration of MCS support was not possible as this information is not captured in HCUP-NIS. Institutional and patient preferences influence MCS device use and associated outcomes and require further evaluation. Factors that can influence outcomes such as angiographic data, PCI location, severity of coronary artery disease, and others were not available in this database. Despite using regression analysis to control for confounders, it is possible that observed results could be due to residual confounding. Finally, our data are only reflective of in-hospital outcomes and we are unable to comment on the long-term outcomes. However, the present study addresses an important and timely topic highlighting the racial disparities in MCS use in a contemporary AMI-CS population.

## 6. Conclusions

Black admissions received MCS support less frequently and had longer length of hospital stay while other races had the highest hospitalization costs. However, despite minor differences in in-hospital mortality among different MCS device categories, overall, we did not identify racial disparities in the in-hospital mortality or acute management in AMI-CS population receiving MCS devices. Future studies with more granular data are essential to establish whether racial differences are truly absent in this population.

## Figures and Tables

**Table 1:** Baseline characteristics of race subgroups of AMI-CS receiving MCS.

Characteristic		White (N = 66,314)	Black (N = 7440)	Others ^a^ (N = 16,317)	*p*
Age (years)		66.6 ± 11.9	63.5 ± 12.6	64.4 ± 12.3	<0.001
Female sex		30.5	39.8	26.7	<0.001
Primary payer	Medicare	56.4	51.4	44.2	<0.001
	Medicaid	7.4	13.0	15.5	
	Private	27.6	24.4	26.4	
	Others ^b^	8.6	11.2	13.8	
Quartile of median household income for zip code	0–25th	25.8	55.1	30.5	<0.001
	26th–50th	28.2	18.9	22.2	
	51st–75th	24.9	14.9	22.9	
	75th–100th	21.1	11.1	24.4	
Charlson Comorbidity Index	0–3	37.7	39.6	39.5	<0.001
	4–6	43.3	39.3	41.0	
	≥7	19.0	21.2	19.5	
Hospital teaching status and location	Rural	4.9	2.1	1.2	<0.001
	Urban non-teaching	28.0	18.7	29.9	
	Urban teaching	67.0	79.1	68.9	
Hospital bed-size	Small	9.9	10.3	10.2	0.03
	Medium	25.8	27.0	26.2	
	Large	64.3	62.6	63.5	
Hospital region	Northeast	18.0	15.6	16.1	<0.001
	Midwest	24.0	20.5	10.5	
	South	39.8	56.2	35.7	
	West	18.2	7.7	37.8	
AMI type	STEMI	67.1	62.3	64.3	<0.001
	NSTEMI	32.9	37.7	35.7	<0.001
Acute organ failure	Respiratory	59.8	57.9	61.2	<0.001
	Renal	46.5	52.8	48.6	<0.001
	Hepatic	15.3	16.1	16.8	<0.001
	Hematologic	19.1	20.3	23.2	<0.001
	Neurologic	19.6	21.6	21.2	<0.001
Cardiac arrest		31.3	31.1	29.9	0.003
Coronary angiography		90.0	88.4	91.4	<0.001
Percutaneous coronary intervention		66.9	65.6	63.7	<0.001
Pulmonary artery catheterization		7.6	7.9	6.9	0.01
Invasive mechanical ventilation		48.8	49.9	54.6	<0.001
Non-invasive ventilation		4.1	4.0	4.5	0.04
Acute hemodialysis		2.1	2.6	3.0	<0.001

Represented as percentages and mean ± standard deviation; ^a^ Hispanic, Asian, Native American, Pacific Islander, and Others; ^b^ Self-Pay, No Charge, Others. Abbreviations: AMI: acute myocardial infarction; CS: cardiogenic shock; MCS: Mechanical circulatory support; NSTEMI: non-ST-segment-elevation myocardial infarction; STEMI: ST-segment-elevation myocardial infarction

**Table 2:** Clinical outcomes of AMI-CS admissions receiving MCS in race sub-groups.

Characteristic		White (N = 66,314)	Black (N = 7440)	Others ^a^ (N = 16,317)	*p*
In-hospital mortality		31.3	31.4	30.2	0.04
Length of stay (days)		10.3 ± 11.2	12.1 ± 14.2	10.9 ± 11.2	<0.001
Palliative care		9.5	8.5	8.2	<0.001
Do-not-resuscitate status		13.2	11.7	12.7	0.002
Total costs (x1000 USD)		242.9 ± 262.9	255.1 ± 262.9	307.2 ± 308.3	<0.001
Durable left ventricular assist device		0.7	0.6	0.7	0.68
Cardiac transplantation		0.1	0.1	0.0	0.30
Discharge disposition	Home	37.0	38.5	42.5	<0.001
	Transfer	14.2	12.6	13.9	
	Skilled nursing facility	30.8	32.4	25.1	
	Home with HHC	17.5	16.0	17.9	
	Against medical advice	0.5	0.5	0.5	

Represented as percentage or mean ± standard deviation; ^a^ Hispanic, Asian or Pacific Islander, Native American, Others. Abbreviations: AMI: acute myocardial infarction; CS: cardiogenic shock; HHC: home health care; MCS: mechanical circulatory support; USD: United States Dollars.

## Data Availability

The data is publicly available via the HCUP-NIS database and hence.

## Supplementary Materials

The following are available online at https://www.mdpi.com/article/10.3390/jcm10071459/s1, Table S1: Administrative codes used for identification of diagnoses and procedures, Table S2: Characteristics of AMI-CS admissions supported with MCS stratified by race and MCS type, Table S3: Predictors of mortality in AMI-CS admissions supported with MCS, Table S4: Clinical outcomes of AMI-CS admissions supported with MCS stratified by race and MCS type.
