# Supplementary material for: Racial Disparities in the Utilization and Outcomes of Temporary Mechanical Circulatory Support for Acute Myocardial Infarction-Cardiogenic Shock
Source: J Clin Med. 2021 Apr 2;10(7):1459. doi: 10.3390/jcm10071459 (PMC8037539; doi:10.3390/jcm10071459)
Supplement: Supplementary file 1 [file jcm-10-01459-s001.pdf]

Supplementary Materials:

Table S1. Administrative codes used for identification of diagnoses and procedures.

Comorbidity	International Classification of Diseases, Clinical Modifications codes
ECMO	ICD-9CM 39.65; ICD-10PCS 5A15223
IABP	ICD-9CM 37.61; ICD-10PCS 5A02110, 5A02210
pLVAD	ICD-9CM 37.68; ICD-10PCS 5A0211D, 5A0221D, 02HA3RJ, 02HA4RJ
Cardiac arrest	427.5
Cardiogenic shock	785.51
Acute kidney injury	584, 584.5, 584.6, 584.7, 584.8, 584.9
Respiratory failure	518.81, 518.82, 518.85, 786.09, 799.1, 96.7, 96.70, 96.71, 96.72
Hepatic failure	570.0, 572.2, 573.3, 573.4
Hematologic failure	286.6-286.9, 287.4, 287.5
Neurological failure	293, 293.0, 293.1, 293.8, 293.81-293.84, 293.89, 293.9, 348.1, 348.3, 348.30, 348.81, 348.39, 780.01, 780.09, 89.14
Invasive hemodynamic assessment	37.21, 37.23, 204
Coronary angiography	37.22, 37.23, 88.53-88.56
Percutaneous coronary intervention	00.66, 36.01, 36.02, 36.05, 36.06, 36.07, 88.57
Invasive mechanical ventilation	96.7, 96.70, 96.71, 96.72
Hemodialysis	39.95

Table S2. Characteristics of AMI-CS admissions supported with MCS stratified by race and MCS type.

Characteristic		IABP (N = 73,979)			pLVAD (N = 9890)			ECMO (N = 1386)			≥MCS (N = 4816)			*P*
		White (N = 54,564)	Black (N = 5971)	Other[a] (N = 13,444)	White (N = 7304)	Black (N = 937)	Other[a] (N = 1649)	White (N = 888)	Black (N = 135)	Other[a] (N = 363)	White (N = 3558)	Black (N = 397)	Other[a] (N = 861)	
**Age (years)**		66.9 ± 11.9	63.8 ± 12.5	64.8 ± 12.3	65.9 ± 11.8	63.3 ± 12.4	63.4 ± 12.3	59.9 ± 10.6	54.2 ± 16.4	58.2 ± 10.8	63.4 ± 10.9	62.6 ± 12.1	62.4 ± 12.3	<0.001
**Female sex**		31.6	40.2	27.3	27.7	42.2	24.3	23.0	34.8	28.1	21.4	30.0	19.7	<0.001
**Primary payer**	**Medicare**	57.5	52.1	44.6	55.1	51.5	48.3	39.2	38.5	26.4	47.0	43.8	37.4	<0.001
	**Medicaid**	7.3	13.4	15.6	7.2	10.8	14.6	10.6	14.8	18.5	7.3	11.3	14.3	
	**Private**	26.7	23.3	25.5	29.0	28.7	23.5	42.6	35.6	44.9	35.9	27.5	38.7	
	**Others[b]**	8.5	11.2	14.2	8.7	9.0	13.6	7.7	11.1	10.2	9.7	17.4	9.6	
**Quartile of median household income for zip code**	**0-25th**	25.5	54.3	31.2	29.3	57.4	30.3	23.3	50.4	12.6	23.4	64.5	27.5	<0.001
	**26th-50th**	28.3	18.3	21.7	29.5	23.2	25.7	26.3	19.3	27.9	24.8	16.8	20.0	
	**51st-75th**	25.1	16.2	22.9	23.3	9.7	22.7	24.9	11.1	22.1	26.0	9.2	23.0	
	**75th-100th**	21.2	11.2	24.2	17.9	9.8	21.2	25.5	19.3	37.4	25.8	9.4	29.5	
**Charlson Comorbidity Index**	**0-3**	37.2	39.3	39.1	38.6	39.5	37.9	51.0	63.0	49.0	41.5	35.3	45.2	<0.001
	**4-6**	43.4	38.8	41.7	42.5	42.4	36.9	42.2	33.3	42.7	43.3	40.8	37.4	
	**≥ 7**	19.4	21.9	19.2	18.9	18.1	25.2	6.8	3.7	8.3	15.2	23.9	17.4	
**Hospital teaching status and location**	**Rural**	5.2	2.4	1.3	4.0	1.6	1.2	0.7	0.0	0.0	3.4	0.0	0.0	<0.001
	**Urban non-teaching**	29.8	19.8	32.5	24.9	20.8	22.7	2.9	0.0	1.7	14.1	4.0	16.3	
	**Urban teaching**	65.0	77.8	66.3	71.1	77.6	76.0	96.4	100.0	98.3	82.5	96.0	83.7	
**Hospital bed-size**	**Small**	10.3	10.0	11.0	9.0	14.5	8.3	1.8	8.1	6.9	6.2	6.8	4.2	<0.001
	**Medium**	26.5	28.0	26.8	25.1	25.8	27.6	15.4	7.4	14.0	19.4	21.9	19.3	
	**Large**	63.1	62.0	62.2	66.0	59.7	64.1	82.8	84.4	79.1	74.3	71.3	76.5	
**Hospital region**	**Northeast**	17.8	15.4	14.9	13.4	14.6	14.6	31.3	27.4	38.3	27.7	17.1	28.0	<0.001
	**Midwest**	24.9	20.6	10.9	18.2	19.9	6.9	23.9	15.6	8.8	21.6	22.4	10.8	
	**South**	39.0	55.5	35.5	49.0	60.1	39.2	34.5	57.0	33.1	34.9	56.4	32.3	
	**West**	18.3	8.5	38.6	19.4	5.4	39.3	10.4	0.0	19.8	15.8	4.0	28.9	
**AMI type**	**STEMI**	67.0	62.0	64.6	65.8	62.2	62.2	75.2	57.0	67.8	68.6	69.3	63.0	<0.001

	**NSTEMI**	33.0	38.0	35.4	34.2	37.8	37.8	24.8	43.0	32.2	31.4	30.7	37.0	<0.001
**Acute organ failure**	**Respiratory**	57.1	55.2	58.6	71.7	68.3	71.2	82.9	68.1	67.5	70.4	71.0	80.1	<0.001
	**Renal**	44.0	50.9	45.8	52.9	53.4	55.8	72.4	72.6	71.3	64.8	73.0	67.9	<0.001
	**Hepatic**	13.3	14.0	15.0	19.6	22.4	19.5	49.1	29.6	36.4	29.8	27.2	31.6	<0.001
	**Hematologic**	17.4	19.3	21.4	21.3	18.2	22.2	44.0	43.0	49.6	33.2	33.0	41.3	<0.001
	**Neurologic**	19.0	21.1	20.7	20.9	20.4	19.5	28.2	48.9	36.9	25.0	21.7	26.6	<0.001
**Cardiac arrest**		30.2	29.0	28.2	32.8	39.9	35.5	41.1	34.1	52.3	43.1	41.8	36.9	<0.001
**Coronary angiography**		91.3	89.5	93.2	90.8	92.3	91.3	32.0	37.8	49.9	81.8	78.8	80.7	<0.001
**PCI**		65.9	63.9	62.3	78.0	77.3	77.7	30.3	25.9	34.2	68.9	78.1	70.3	<0.001
**PAC**		7.3	7.2	6.7	7.8	8.6	6.4	8.1	12.6	8.8	11.5	15.6	11.7	<0.001
**IMV**		47.7	49.0	53.7	53.5	51.9	57.0	66.1	45.2	69.1	52.2	61.0	57.7	<0.001
**Non-invasive ventilation**		4.3	4.5	4.8	3.8	2.9	3.2	1.2	0.0	0.0	2.2	0.0	3.7	<0.001
**Acute hemodialysis**		2.0	2.6	3.1	2.3	2.1	3.0	5.1	7.4	2.8	3.5	1.3	1.7	<0.001

Legend: Represented as percentage; [a]Hispanic, Asian or Pacific Islander, Native American, Others; [b]Self-Pay, No Charge, Others. **Abbreviations:** AMI: acute myocardial infarction; CS: cardiogenic shock; ECMO: extracorporeal membrane oxygenation; IABP: intraaortic balloon pump; IMV: invasive mechanical ventilation; MCS: mechanical circulatory support; NSTEMI: non-ST-segment-elevation myocardial infarction; PAC: pulmonary artery catheterization; PCI: percutaneous coronary intervention; pLVAD; percutaneous left ventricular assist device; STEMI: ST-segment-elevation myocardial infarction.

Table S3. Predictors of mortality in AMI-CS admissions supported with MCS.

Total cohort (N = 90,071)		Odds ratio	95% confidence interval		*P*
			Lower Limit	Upper Limit	
Race	White	Reference category			
	Black	0.98	0.93	1.05	0.60
	Others[a]	0.96	0.92	1.01	0.10
Age (years)	<75 years	Reference category			
	>75 years	1.48	1.42	1.55	<0.001
Female sex		1.12	1.08	1.16	<0.001
Primary payer	Medicare	Reference category			
	Medicaid	0.64	0.60	0.68	<0.001
	Private	0.62	0.59	0.65	<0.001
	Others[b]	0.91	0.86	0.97	0.004
Quartile of median household income for zip code	0-25th	Reference category			
	26th-50th	0.89	0.86	0.94	<0.001
	51st-75th	0.81	0.78	0.85	<0.001
	75th-100th	0.77	0.74	0.81	<0.001
Hospital teaching status and location	Rural	Reference category			
	Urban Non-Teaching	0.91	0.83	0.99	0.03
	Urban Teaching	0.97	0.89	1.06	0.51
Hospital bed-size	Small	Reference category			
	Medium	1.10	1.03	1.17	0.003
	Large	1.13	1.06	1.19	<0.001
Hospital region	Northeast	Reference category			
	Midwest	0.81	0.77	0.86	<0.001
	South	0.99	0.95	1.04	0.84
	West	0.92	0.87	0.97	0.002
Charlson Comorbidity Index	0-3	Reference category			
	4-6	1.04	1.00	1.09	0.04
	≥ 7	0.95	0.90	1.00	0.05
Type of AMI	STEMI	Reference category			

	**NSTEMI**	0.75	0.72	0.78	<0.001
**Acute organ failure**	**Respiratory**	1.49	1.43	1.55	<0.001
	**Renal**	1.40	1.35	1.45	<0.001
	**Hepatic**	1.58	1.51	1.65	<0.001
	**Hematologic**	0.90	0.85	0.93	<0.001
	**Neurologic**	1.05	1.01	1.09	0.02
**Out of hospital cardiac arrest**		2.26	2.18	2.34	<0.001
**Coronary angiography**		0.70	0.66	0.73	<0.001
**Percutaneous coronary intervention**		0.93	0.90	0.97	<0.001
**Pulmonary artery catheterization**		1.05	0.99	1.12	0.11
**Invasive mechanical ventilation**		1.66	1.60	1.72	<0.001
**Acute hemodialysis**		1.51	1.37	1.67	<0.001
**Do-not-resuscitate status**		8.18	7.79	8.60	<0.001

Legend: [a]Hispanic, Asian or Pacific Islander, Native American, Others; [b]Self-Pay, No Charge, Others. **Abbreviations:** AMI: acute myocardial infarction; CS: cardiogenic shock; MCS: mechanical circulatory support; NSTEMI: non-ST-segment elevation myocardial infarction; STEMI: ST-segment elevation myocardial infarction.

Table S4. Clinical outcomes of AMI-CS admissions supported with MCS stratified by race and MCS type.

Characteristic		IABP (N = 73,979)			pLVAD (N = 9890)			ECMO (N = 1386)			≥MCS (N = 4816)			*P*
		**White (N = 54,564)**	**Black (N = 5971)**	**Other[a] (N = 13,444)**	**White (N = 7304)**	**Black (N = 937)**	**Other[a] (N = 1649)**	**White (N = 888)**	**Black (N = 135)**	**Other[a] (N = 363)**	**White (N = 3558)**	**Black (N = 397)**	**Other[a] (N = 861)**	
**In-hospital mortality**		28.1	27.3	26.8	43.2	44.2	41.8	54.1	73.3	60.9	48.8	48.6	48.8	<0.001
**Length of stay (days)**		9.9 ± 9.5	11.9 ± 14.1	10.8 ± 10.6	9.5 ± 10.7	10.5 ± 11.9	9.3 ± 11.1	21.9 ± 29.5	13.6 ± 12.5	13.5 ± 14	14.8 ± 20.7	17.7 ± 19.2	15.7 ± 16.6	<0.001
**Palliative care**		8.6	7.9	7.2	12.9	7.5	10.1	22.2	26.7	19.6	13.0	14.4	16.0	<0.001
**Do-not-resuscitate status**		12.6	10.9	12.4	16.0	15.4	14.5	17.0	23.0	9.9	14.8	11.3	15.4	<0.001
**Total costs (x1000 USD)**		210 ± 201	224 ± 231	275 ± 259	315 ± 228	332 ± 291	358 ± 293	667 ± 763	503 ± 486	481 ± 469	488 ± 527	461 ± 354	615 ± 605	<0.001
**Durable LVAD**		0.3	0.4	0.3	0.8	1.1	1.6	14.9	3.7	2.8	3.8	2.5	3.5	<0.001
**Cardiac transplantation**		0.0	0.1	0.0	0.1	0.0	0.0	0.7	0.0	0.0	0.3	0.0	0.6	<0.001
**Discharge disposition**	**Home**	38.4	39.6	44.4	34.2	38.2	36.8	14.0	0.0	19.7	17.5	20.6	19.5	
	**Transfer**	12.9	12.9	12.4	17.4	8.7	20.2	25.0	27.8	26.8	31.8	14.7	30.4	
	**SNF**	30.4	30.7	24.5	32.9	42.3	25.1	43.9	41.7	24.6	33.3	42.6	38.8	<0.001
	**Home/HHC**	17.8	16.4	18.3	15.4	9.8	16.4	17.2	30.6	28.9	17.2	22.1	10.2	
	**AMA**	0.5	0.5	0.4	0.1	1.0	1.6	0.0	0.0	0.0	0.3	0.0	1.1	

Legend: Represented as percentage or mean ± standard deviation; [a]Hispanic, Asian or Pacific Islander, Native American, Others. **Abbreviations:** AMA: against medical advice; AMI: acute myocardial infarction; CS: cardiogenic shock; ECMO: extracorporeal membrane oxygenation; HHC: home health care; IABP: intraaortic balloon pump; LVAD: left ventricular assist device; MCS: mechanical circulatory support; pLVAD; percutaneous left ventricular assist device; SNF: skilled nursing facility; USD: United States Dollars.
